# Yeast Species in the Oral Cavities of Older People: A Comparison between People Living in Their Own Homes and Those in Rest Homes

**DOI:** 10.3390/jof5020030

**Published:** 2019-04-12

**Authors:** Nurulhuda Mohd Thiyahuddin, Erwin Lamping, Alison M. Rich, Richard D. Cannon

**Affiliations:** Sir John Walsh Research Institute, Faculty of Dentistry, University of Otago, Dunedin 9054, New Zealand; nurul.thiyahuddin@otago.ac.nz (N.M.T.); erwin.lamping@otago.ac.nz (E.L.); alison.rich@otago.ac.nz (A.M.R.)

**Keywords:** oral candidiasis, older people, elderly, rest home, yeast carriage, *Candida albicans*

## Abstract

Oral candidiasis is prevalent among older people due to predisposing factors such as impaired immune defenses, medications and denture use. An increasing number of older people live in rest home facilities and it is unclear how this institutionalized living affects the quantity and type of fungi colonizing these people’s oral cavities. Smears and swabs of the palate and tongue and saliva samples were taken from participants residing in rest homes (RH; *n* = 20) and older people living in their own homes (OH; *n* = 20). Yeast in samples were quantified and identified by culturing on CHROMagar *Candida* and sequencing the ITS2 region of rDNA. A higher proportion of RH residents had *Candida* hyphae present in smears compared to OH participants (35% vs. 30%) although this difference was not statistically significant (*p* = 0.74). RH residents had, on average, 23 times as many yeast per mL saliva as OH participants (*p* = 0.01). Seven yeast species were identified in OH samples and only five in RH samples, with *Candida albicans* and *Candida glabrata* being the most common species isolated from both participant groups. The results indicate that older people living in aged-care facilities were more likely to have candidiasis and have a higher yeast carriage rate than similarly aged people living at home. This may be due to morbidities which led to the need for residential care and/or related to the rest home environment.

## 1. Introduction

The oral cavity is home to many hundreds of species of microorganisms. The majority of these microbes are present in biofilms on the various oral surfaces. The immune systems of healthy individuals maintain stable biofilm communities that help prevent oral infections. When immune defenses are impaired this can cause dysbiosis of the microbiota and lead to bacterial, viral or fungal infections.

*Candida* species are a normal component of the oral microbiota [1] that are carried in the mouths of about 40–60% of healthy individuals [2]. While *Candida albicans* is the most prevalent species [3,4], non-*albicans Candida* species that can be found in healthy individuals include *Candida glabrata, Candida tropicalis, Candida parapsilosis,* and *Candida krusei* [5]. Other species such as *Candida guilliermondii, Candida kefyr, Candida rugosa, Candida dubliniensis* and *Candida famata* have also been reported, but less frequently [6,7]. Oral candidiasis is the most common type of fungal infection of the oral cavity and may present as pseudomembranous candidiasis, erythematous candidiasis, plaque-like/nodular candidiasis, denture-associated candidiasis, angular cheilitis, median rhomboid glossitis and chronic mucocutaneous candidiasis [8]. Due to its prevalence as a commensal microbe, *C. albicans* is the most frequent cause of oral candidiasis [9]. A preliminary clinical diagnosis of candidiasis can be made based on the medical history of the individual, their symptoms and an oral examination. This diagnosis can be confirmed histologically by the presence of hyphae in mucosal smears. Patients with oral candidiasis can be treated with antifungal agents such as polyenes (e.g., amphotericin B and nystatin) and azole derivatives (e.g., fluconazole, ketoconazole, clotrimazole and miconazole). It has been reported that 20% of patients with oral candidiasis experience recurrence of infection, and around 30% of the recurrences were caused by *Candida* strains different from the first episode of infection [10]. *Candida* species differ in their susceptibility to antifungal agents and *C. glabrata* and *C. krusei* show reduced susceptibility to azoles [4,11] and *Candida lusitaniae* can develop resistance to amphotericin B [12]. In addition, *C. parapsilosis* has been reported to cause outbreaks of candidemia in immunocompromised patients in hospital settings [13] where it is thought to be transmitted by the hands of health care workers. Therefore, in institutionalized settings it is important to know what species of *Candida* are colonizing individuals.

Colonization of the oral cavity by *Candida* depends on the entry of yeast cells, attachment and growth, and cell removal from the oral cavity—the rate of yeast replication must at least match the rate of clearance [1]. Key factors that predispose for *Candida* colonization include systemic factors caused by drug therapy (e.g., broad spectrum antibiotics, corticosteroids), reduced saliva flow, chronic disease processes, malnutrition, immunosuppression and neoplasia while local factors include wearing dentures overnight, ill-fitting dentures and poor prosthesis hygiene [14]. In the oral cavity, components of the innate immune system, such as salivary flow and biological components within saliva, play a major role in preventing infections. Factors that reduce saliva flow predispose to infections such as candidiasis. In studies of people with xerostomia (dry mouth) it has been shown that there is an inverse correlation between salivary flow rate and the number of *Candida* cells in oral samples [15,16]. There was also an inverse correlation between the salivary flow rate and the severity of oral candidiasis [15]. The proportion of the population receiving medications increases with age and older people are often prescribed multiple medications, many of which reduce saliva flow rates. Denture wearing also increases with age and dentures obstruct saliva flow across mucosal surfaces reducing the clearance of microbes and creating environments that promote fungal growth. Therefore, it is not surprising that older people are more likely than younger people to have oral mucosal lesions, including candidiasis [17].

Around the world, populations are ageing. The WHO estimates that by 2050 the proportion of the population that is ‘older’ (in this case above the age of 60) will have increased from 12% in 2015 to 22%, that is, from 900 million to 2 billion people [18]. Studies have shown elderly patients living in institutional care facilities, such as nursing homes and hospitals, are more susceptible to oral and systemic *Candida* infections [19], mainly due to comorbidities, in-dwelling devices and common care facility processes. What is concerning is the emergence of non-*albicans Candida* strains with lower antifungal susceptibility in self-caring, nursing home residents [4]. *C. glabrata* and *C. krusei* strains showed reduced susceptibility to azoles and *C. rugosa* and *C. krusei* strains were less susceptible to flucytosine than other yeast. The presence of *Candida* strains with reduced susceptibility to antifungal agents could pose a problem for older people if they become immune compromised and develop candidiasis.

A significant number of older people live in residential aged care facilities [20], which in New Zealand was approximately 32,000 people in 2013 [21]. The objective of this study was to compare the *Candida* species colonizing the oral cavities of people living in rest homes with those living in their own homes.

## 2. Materials and Methods

### 2.1. Participants

This pilot cross-sectional study was a convenience sampling of two groups of older people; people living at rest home facilities (*n* = 20) and people living in their own homes (*n* = 20). Rest home participants came from either Montecillo Veterans Home and Hospital, or Little Sisters of the Poor, both located in Dunedin, New Zealand. Participants for the control group were members of the public recruited from posters circulated via Age Concern, Bowling Clubs Dunedin and personal contact. Inclusion criteria were that participants had to be above the age of 65, healthy or have well controlled systemic disease, have sufficient mouth opening to allow intraoral access and able to give consent. Participants were excluded from taking part in this study if they had used antibiotics or antifungals in the past 2 months, were terminally ill or were current smokers. Ethical approval for this study was obtained from the University of Otago Human Ethics Committee (approval number H17/081).

### 2.2. Clinical Examination

A clinical examination was carried out by direct observation of the condition of the oral mucosa. Participants who exhibited mucosal inflammation and had hyphae present in the smear test were prescribed antifungal agents. If they wore dentures they were advised to leave them out at night and denture hygiene instructions were given. The rest home in-house medical doctor and nurse manager were involved in this process to further support the participants.

### 2.3. Collection of Oral Samples

Unstimulated saliva was collected from participants. Participants were requested to allow their saliva to pool in their mouths for one minute then expectorate it into a glass measuring cup. This was done 5 times. The total volume of saliva produced was recorded and the saliva transferred to a sterile 15 mL Falcon tube. Patients who were unable to generate saliva rinsed their mouths with 2 mL of sterile water. This was to enable analysis of salivary gland function and the yeast present in saliva. Participants’ palates and tongues were swabbed with sterile cotton buds and each swab was placed in 2 mL sterile water in a sterile 15 mL Falcon tube.

Smears of the palate and tongue were taken by gently rubbing a wooden spatula on the dorsal surface of the tongue and hard palate, avoiding the area that had just been swabbed, but immediately adjacent to it. The material obtained on the spatula was smeared onto a glass slide and the slides immersed in Coplin jars filled with 95% ethanol. The Coplin jars were transported to the University of Otago Faculty of Dentistry Oral Pathology Centre where they were stained with periodic acid-Schiff (PAS).

### 2.4. Analysis of Samples

Saliva and mucosal swabs were transported on ice to the Faculty of Dentistry Molecular Biosciences Laboratory for analysis and storage (4 °C). Whole saliva samples and suspensions of palate and tongue swabs were vortexed for 30 s and portions (100 µL) were spread on CHROMagar *Candida* agar plates (Becton Dickinson & Co., Franklin Lakes, NJ, USA) which were incubated at 37 °C for 48 h. *Candida* species were presumptively identified according to colony color, and colony forming units (cfu) per ml sample were determined. When the numbers of cfu on plates were too many to count, portions of the stored yeast suspensions were diluted in sterile water 100- or 1000-fold and plated and incubated as described above. The numbers of colonies on plates were converted to cfu/mL saliva by multiplying by the dilution factor and then by 10 (as 100 µL was plated). Yeast species were confirmed by colony polymerase chain reaction (PCR) amplification and sequencing of the rDNA internal transcribed spacer region ITS2 [22]. Colony PCR was carried out using KOD FX Neo DNA Polymerase (Toyobo, Osaka, Japan), where each 20 µL PCR reaction contained 0.6 µL sterile water, 10 µL 2 × PCR Buffer for KOD FX Neo, 4 µL dNTPs (2 mM), 2 µL each of forward and reverse primers ITS3(F) 5′-GCATCGATGAAGAACGCAGC and ITS4(R) 5′-TCCTCCGCTTATTGATATGC [23] (3.2 µM), 1 µL of template cells (suspended in 20 µL sterile water) and 0.4 µL KOD FX Neo (1.0 U/µL). The PCR cycle conditions were: initial denaturation at 98 °C for 30 s (1 cycle); followed by 44 cycles of the following steps: denaturation at 98 °C for 10 s; annealing at 55 °C for 10 s; elongation at 68 °C for 40 s; and final elongation step at 68 °C for 1 min.

The presence of PCR products was confirmed by running 1 µL of colony PCR sample with 5 µL of 1× loading dye on agarose gel (1.5% *w*/*v*) through electrophoresis at 100 V for 28 min with ethidium bromide staining. DNA oligomer primers were removed from colony PCR products with ExoSAP-IT™ (ThermoFisher Scientific, Christchurch, New Zealand) according to the manufacturer’s instructions. DNA sequencing was carried out using primer ITS3(F) by the Genetic Analysis Service, Anatomy Department, University of Otago. Results were analysed with the computer programs FinchTV (version 1.5.0) and UniPro UGENE (version 1.29.0) and the sequences compared with the publicly available database at https://www.ncbi.nlm.nih.gov/BLAST/.

PAS-stained slides were examined by a consultant oral pathologist to determine the presence or absence of fungal hyphae.

### 2.5. Statistical Analysis

The *t*-test was used to compare parametrically distributed data. The Mann-Whitney *U* test was used to compare non-parametrically distributed data. Differences were considered significant if *p* < 0.05. For binary dependent variables, SPSS software (version 25, IBM Corporation) was used for cross-tabulations, with Chi-square tests used to determine statistical significance (at the 0.05 level).

## 3. Results

### 3.1. Participant Demographics

The participants in the two groups (those who lived in their own homes, and those who lived in rest homes) were sex-matched and although the participants from rest homes were, on average, 2.9 years older than those who lived in their own homes, this difference was not statistically significant (Table 1).

The participants from rest homes were more likely to wear dentures (full or partial) than those living in their own homes, and they had, on average, a slightly lower salivary flow rate, although this difference was not statistically significant (Table 1). Fifty-five percent of the rest home participants had salivary gland hypofunction (defined as an unstimulated whole salivary flow rate < 0.2 mL/min [24]) as did 40% of those living at home, this difference was not statistically significant (*p* = 0.34, chi-square test). Rest home participants were more likely to be diagnosed with candidiasis (hyphae present in smears of the palate and tongue; Table 1) than participants living in their own homes (difference not statistically significant; *p* = 0.74 Table 1).

The participants were taking a variety of, and usually multiple, medications. The number of medications being taken by people living in their own homes ranged from 0 to 11 with a mean of 5.4 medications (Table 1). Rest home participants were taking, on average, more medications (6.6, range: 1–12), but this difference was not statistically significant (*p* = 0.25; *t*-test). If, however, all participants were categorized as either having a normal salivary flow rate or a low salivary flow rate (<0.2 mL/min), those with an abnormally low flow rate were taking, on average, significantly more medications than those with a normal flow rate (7.4 vs. 4.8; *p* = 0.005, *t*-test).

### 3.2. Colonization with Yeast

**Figure 1:**
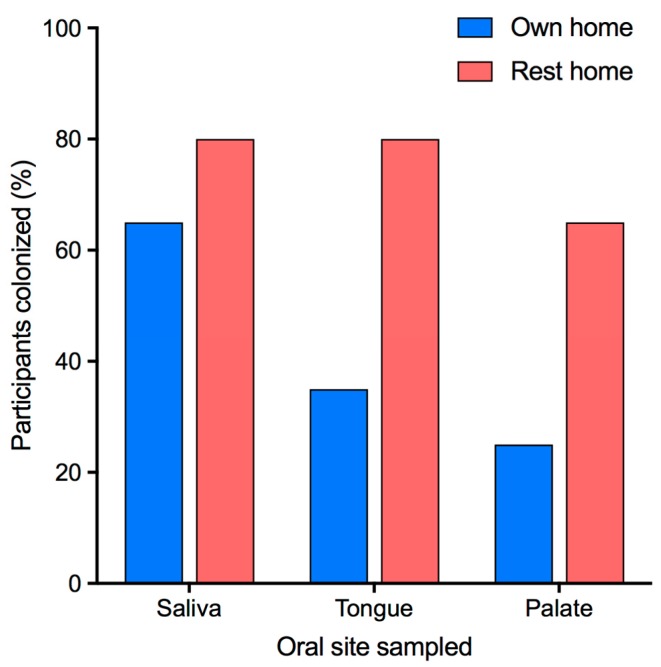
Presence of yeast at oral sites in participants. Percentage of participants colonized by yeast at each oral site.

Eighty percent of the rest home participants were colonized by yeast at one or more of the oral sites sampled (saliva, tongue, or palate) as were 65% of participants living in their own homes. Colonization rates at the three sites varied for both groups of participants (Figure 1).

**Figure 2:**
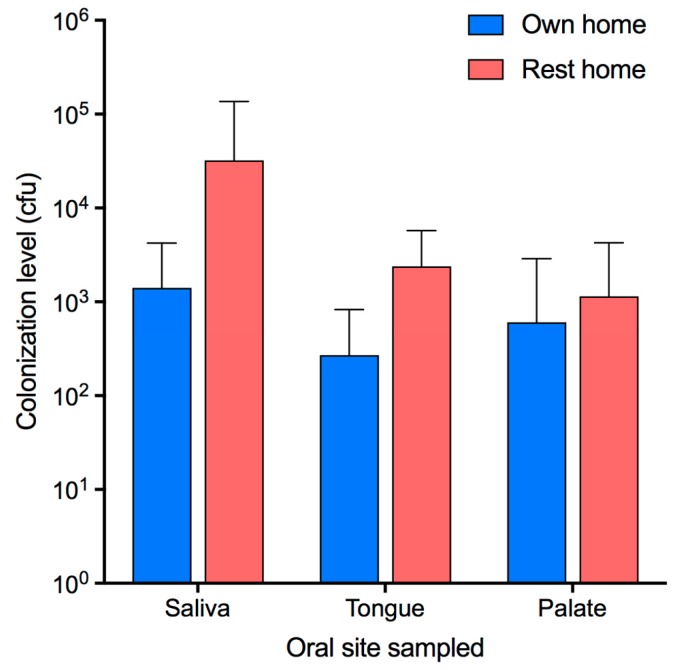
Quantity of yeast at oral sites in participants. Level of yeast colonization at each site in each group (cfu/mL of saliva samples, cfu/swab for tongue and palate swabs ± standard deviation).

Of the oral samples, saliva samples were most commonly yeast-positive followed by tongue swabs and then palate swabs. Each oral site was more often colonized by yeast in participants from rest homes. This increased colonization of rest home participants was statistically significant for all three sites (saliva *p* = 0.01, tongue *p* = 0.001, palate *p* = 0.03; Table 1). Eighty-four percent of those wearing dentures had saliva that contained yeast whereas saliva was yeast-positive in only 53% of those without dentures. This difference was statistically significant (*p* = 0.04; chi-square test). The numbers of yeast in each sample differed considerably and were higher in those participants with PAS-positive smears. Of the 13 participants with PAS-positive smears, 11 had yeast detected in either the tongue or palate swab samples. Yeast were also detected in either the tongue or palate swab samples in a further 11 participants who were not PAS-positive. For those people colonized with yeast, the mean number of cfu per ml of saliva was 40,305 (median: 4425; range: 410–472,000) for participants from rest homes, whereas the mean for participants living in their own homes was only 2171 (median: 230; range: 20–9160). As several of the participants living in their own homes had no detectable yeast in their saliva the mean colonization level for the group was 1410 cfu/mL (median: 50 cfu/mL) whereas it was 32,240 cfu/mL (median: 2260 cfu/mL) for the rest home group (Table 1, Figure 2). The numbers of yeast in each type of samples from participants from rest homes were significantly higher than those from people living in their own homes (Table 1).

### 3.3. Species of Colonizing Yeast

**Figure 3:**
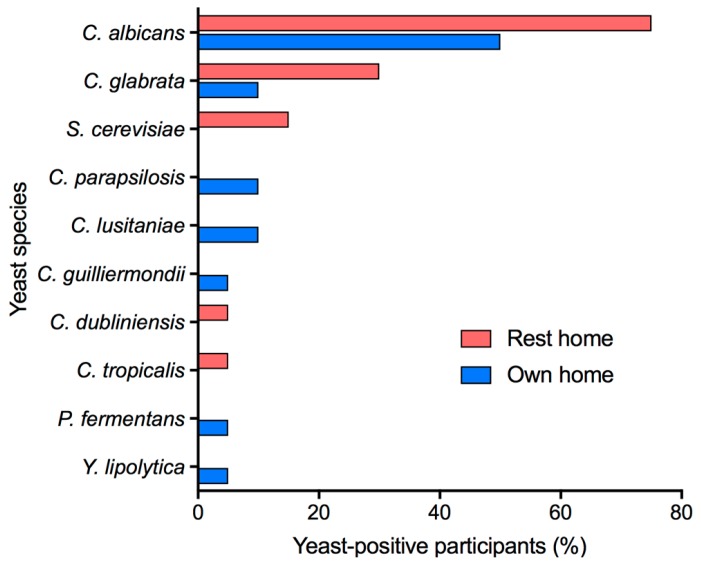
Species of yeast colonizing participants.

Yeast were identified by sequencing their rDNA internal transcribed spacer region ITS2. The yeast species most frequently isolated in each sample type from both participant groups was *C. albicans* followed by *C. glabrata*. Seven yeast species were identified in samples from individuals living in their own homes: *C. albicans, C. glabrata, C. parapsilosis, C. lusitaniae, C. guilliermondii, Pichia fermentans* and *Yarrowia lipolytica* (Figure 3). Only five yeast species (*C. albicans, C. glabrata, Saccharomyces cerevisiae, C. dubliniensis* and *C. tropicalis*) could be identified in rest-home participants, even though more samples were yeast-positive.

**Figure 4:**
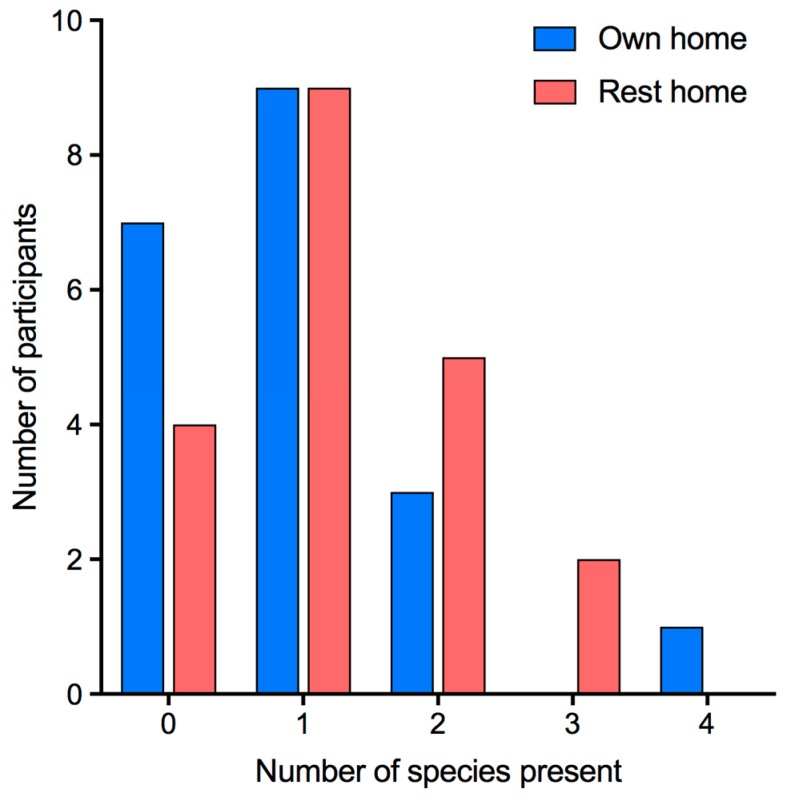
Number of yeast species colonizing individual participants.

Of the 60 oral samples taken from the participants living in their own homes, 25 were positive for yeast, whereas 45 of the 60 residential home samples contained yeast. Of the total 70 oral samples that were yeast-positive, 42 contained only one species of yeast and 28 had more than one species. The species that were found together most frequently were *C. albicans* and *C. glabrata.* The largest number of species found in the oral cavity of a participant (who lived in their own home) was four (Figure 4).

## 4. Discussion

In this study the types, and quantities, of yeast colonizing the oral surfaces of older people living in rest homes were compared with those in people living in their own homes. Three oral sites were sampled to make these measurements, the tongue, the palate, and whole unstimulated saliva. The tongue was sampled as it has been shown to be one of the most frequently colonized oral sites [25], and the palate is also often colonized in patients with full upper dentures [5]. We found that in both groups of participants the tongue was more frequently colonized than the palate, and that yeast were most frequently found in saliva; all individuals with yeast on their tongues or palate also had yeast in saliva, and for some individuals yeast were only detected in their saliva. This is probably because if yeast colonize oral surfaces, they are likely to be washed off to some extent by saliva. Thus, for a study of the variety of fungi in people’s mouths, yeast in saliva can be used as a proxy for oral colonization and saliva may act as a reservoir for colonization of other oral sites. It is difficult to assign a concentration of yeast in saliva, on swabs, or in smears that might indicate candidiasis as host factors are important in disease progression. The presence of hyphae in smears is a good indication of clinically-significant disease as the hyphal form can penetrate tissues. However, many *Candida* species, such as *C. glabrata,* do not form hyphae making the diagnosis of candidiasis from the inspection of smears more difficult. The fact that yeast were cultured from more people than had PAS-positive smears may reflect the greater sensitivity of culturing yeast from swabs or saliva samples, but this positive culture does not give the clinician an indication of the disease status.

There are a number of factors that are known to favor oral colonization with yeast. These include reduced salivary flow rates [16,26] and wearing dentures [27]. In the present study we found that people living in their own homes who had a low unstimulated salivary flow rate (<0.2 mL/min) had, on average, a greater concentration of yeast in their saliva than people with a higher flow rate (2675 cfu/mL compared to 568 cfu/mL, *p* = 0.009; Mann–Whitney *U* test). This was not the case for rest-home residents. Although a larger proportion of rest-home residents who had a normal salivary flow rate were yeast-negative compared to those with reduced flow rates (33% versus 9%) their mean colonization level was higher than that for residents with a reduced salivary flow rate due to two residents with high salivary yeast concentrations and normal flow rates. Unstimulated saliva flow rates reduce with age [28] and older people are often prescribed medications which are known to be xerogenic (cause dry mouth). Due to the number and variety of medications our participants were prescribed, it was not possible to control for medications between the groups. There was no significant difference in the mean number of medications taken by the two groups of participants, but there was a significant association between low salivary flow rate and a higher number of medications. Participants in both groups who wore dentures had greater concentrations of yeast in their saliva than those participants who did not wear dentures, but the differences were not statistically significant.

CHROMagar *Candida* is a useful medium for the presumptive identification of *C. albicans* from biological samples and for distinguishing other fungal species. Combining this primary screen with rDNA ITS2 sequencing of isolates enabled precise identification of the predominant fungal species present in oral samples without the detection of extremely minor components of the mycobiome as is often the case with direct next generation sequencing of biological samples. As has been shown previously [4,5,16], the most common yeast species found in the mouths of participants was *C. albicans.* The second most frequently isolated species was *C. glabrata*. This may reflect the increased prevalence of this species in clinical samples that has been reported in recent years [29]. It is of concern that of the 10 yeast species detected in participants, seven (*C. albicans*, *C. glabrata*, *C. parapsilosis*, *C. lusitaniae*, *C. guilliermondii*, *C. tropicalis,* and *Y. lipolytica*) have been reported as being potentially resistant to antifungals [12,29,30,31,32,33]. It was beyond the scope of the present project to measure the antifungal susceptibility of all the fungal isolates, but this would provide an indication of whether the older adults sampled were at risk from a fungal infection that would be difficult to treat.

As populations age, an increasing number of older people are being cared for in rest homes, yet there are few studies on the microbial colonization of people living in these institutions. One comparison of older people in institutionalized long-term care with those not in such care found that oral colonization with yeast was more frequent in the institutionalized individuals [34]. This study did not, however, identify the colonizing yeast. We found that oral sites were more frequently colonized by yeast in rest-home participants than in people living in their own home and that the mean concentration of yeast in the saliva of rest-home participants was 23 times higher than that in non-rest home participants (the median was 45 times higher). This may indicate poorer oral hygiene in the rest home participants which may be due to the reason they are in rest homes—they are less capable of independent living. The increased numbers of opportunistic fungal pathogens in rest home residents suggests that the rest homes would be well advised to pay greater attention to the oral hygiene of residents.

It was interesting to note that a greater variety of yeast species was detected in people living in their own homes (7 species) compared to those living in rest homes (5 species) with only two species (*C. albicans* and *C. glabrata*) common to both groups. A possible explanation is that communal living and shared care assistants results in the sharing of yeast between rest home residents. This possibility could be investigated further by MLST (multilocus sequence typing) analysis of the predominant species in both groups, *C. albicans*, to determine the relatedness of strain types in the two groups, as we have done previously for people wearing dentures [35]. Alternatively, a higher proportion of people in rest homes may have morbidities that favor yeast growth, and yeast species particularly well-adapted to the oral environment in these people may out-compete other species. This could lead to high numbers of a single yeast species in such individuals. Species with the potential to be drug resistant were present in both the rest home residents (*C. albicans*, *C. glabrata*, *C. dubliniensis*, and *C. tropicalis*) and people living in their own homes (*C. albicans, C. glabrata, C. parapsilosis*, *C. lusitaniae*, *C. guilliermondii*, and *Y. lipolytica*) emphasizing the need for all older people to keep in good health and maintain good oral hygiene.

This study has some limitations. One is the small sample size. While a few studies report the colonization of older people living in institutionalized care by yeast [34,36], there are no quantitative analyses of the different yeast present. Thus, it was not possible to perform a sample-size estimation for this study, and a pilot investigation was undertaken. Also, participants were recruited from two rest homes which introduced a potential confounder. Despite the small sample size, the present study revealed some important statistically significant differences between the oral colonization of people living in rest homes and those living in their own homes. The results from this study, therefore, provide a justification, and basis, for examining the oral fungal colonization of larger samples of older people from a single rest home and older people living in their own homes.

## Figures and Tables

**Table 1:** The demographics, clinical features, and colonization status of the participants.

Variable	Participant Location		*p* Value
	Own Home (*n* = 20)	Rest Home (*n* = 20)	
Gender			
Female	13	13	
Male	7	7	
Mean age (range)	83.1 (71–92)	86.0 (72–94)	0.50 ^a^
Proportion with dentures	55%	70%	0.33 ^b^
Mean salivary flow rate [mL/min] (range)	0.25 (0–0.7)	0.23 (0–0.6)	0.35 ^a^
Proportion with hyphae in smears	30%	35%	0.74 ^b^
Mean number of medications (range)	5.4 (0–11)	6.6 (1–12)	0.25 ^a^
Proportion with saliva colonized by yeast	65%	80%	0.29 ^b^
Proportion with tongue colonized by yeast	35%	80%	**0.004** ^b^
Proportion with palate colonized by yeast	25%	65%	**0.01** ^b^
Level of yeast colonization of saliva (cfu/mL)			
Mean	1410	32,240	**0.01** ^c^
Median (range)	50 (0–9160)	2260 (0–4.72 × 10^5^)	
Level of yeast colonization of tongue (cfu/swab)			
Mean	271	2400	**0.001** ^c^
Median (range)	0 (0–2200)	1970 (0–14,760)	
Level of yeast colonization of palate (cfu/swab)			
Mean	606	1150	**0.03** ^c^
Median (range)	0 (0–10,240)	200 (0–14,100)	

^a^*t*-test; ^b^ Chi-square test; ^c^ Mann–Whitney *U* test. *p* values in bold typeface are significant (<0.05).
